# Nanoparticle-antagomiR based targeting of miR-31 to induce osterix and osteocalcin expression in mesenchymal stem cells

**DOI:** 10.1371/journal.pone.0192562

**Published:** 2018-02-14

**Authors:** Mark McCully, João Conde, Pedro V. Baptista, Margaret Mullin, Matthew J. Dalby, Catherine C. Berry

**Affiliations:** 1 Centre for Cell Engineering, University of Glasgow, Glasgow, Lanarkshire, United Kingdom; 2 Massachusetts Institute of Technology, Institute for Medical Engineering and Science, Harvard-MIT Division for Health Sciences and Technology, Cambridge, Massachusetts, United States of America; 3 UCIBIO, DCV, Faculdade de Ciencias e Tecnologia, Universidade Nova de Lisboa, Campus de Caparica, Caparica, Portugal; 4 Electron Microscopy Unit, University of Glasgow, Glasgow, Lanarkshire, United Kingdom; Helsingin Yliopisto, FINLAND

## Abstract

Mesenchymal stem cells are multipotent adult stem cells capable of generating bone, cartilage and fat, and are thus currently being exploited for regenerative medicine. When considering osteogenesis, developments have been made with regards to chemical induction (e.g. differentiation media) and physical induction (e.g. material stiffness, nanotopography), targeting established early transcription factors or regulators such as runx2 or bone morphogenic proteins and promoting increased numbers of cells committing to osteo-specific differentiation. Recent research highlighted the involvement of microRNAs in lineage commitment and terminal differentiation. Herein, gold nanoparticles that confer stability to short single stranded RNAs were used to deliver MiR-31 antagomiRs to both pre-osteoblastic cells and primary human MSCs in vitro. Results showed that blocking miR-31 led to an increase in osterix protein in both cell types at day 7, with an increase in osteocalcin at day 21, suggesting MSC osteogenesis. In addition, it was noted that antagomiR sequence direction was important, with the 5 prime reading direction proving more effective than the 3 prime. This study highlights the potential that miRNA antagomiR-tagged nanoparticles offer as novel therapeutics in regenerative medicine.

## Introduction

Bone marrow-derived mesenchymal stem cells (MSCs) can both self-renew and are multipotent, capable of differentiation down multiple skeletal lineages, including osteoblasts, chondrocytes and adipocytes. These characteristics are key in current and future MSC-based therapeutics, particularly in orthopaedics, and are the driving force behind research on understanding the regulation of differentiation [1, 2]. To date, there are a number of critical signaling pathways which have been identified as being involved in regulating MSC lineage commitment, the most established of these include Wnt, Hedgehog, Notch and bone mophogenic protein (BMP) signaling; all of which target runx2, a master osteogenic transcription factor [3, 4]. Recent research has turned towards additional regulators of MSC differentiation. The discovery of microRNAs as a mechanism for regulating gene expression in the early 2000s has opened up a new avenue of study in this regard [5].

MicroRNAs (miRNAs or miRs) are small, single stranded RNA molecules approximately 20 nucleotides long, involved in the RNA interference (RNAi) pathway [5]. Before being cleaved into single strands, miRs exist as a stem loop with both a guide strand (5’ prime arm) and passenger strand (3’ prime arm). The differences between the activity of the miRs strands is still an active area of debate and research. Here we describe a clear difference in the action between the guide strand (5’) and the passenger strand (3’). MiRs, unlike short interfering RNAs (siRNAs), do not bind with complete complementarity to targeted RNA sequences. This lack of complementarity allows miRs to bind and reduce the expression of a number of mRNA transcripts, thus offering an attractive mechanism for broad attenuation of target genes [6]. In 2006, Thompson *et al* performed the first global analysis of miR levels. Mature miRs were analysed and showed widespread post-transcriptional regulation of mRNA [7], regulating a wide spectrum of biological processes from differentiation, [8, 9] to tumorigenesis [10]; therefore miRs have increasingly become an exciting potential target for future therapeutics.

It is becoming increasingly evident that miRs play a critical role in regulation of MSC growth, osteogenic lineage commitment and terminal differentiation, as indicated in Table 1 [11].

**Table 1:** The role of miRs in several bone cell types.

Bone Cell Type	Cell Role	miR Involved
MSCs	Proliferation and homeostasis	miR-135, miR-138, miR-23a, miR-30c, miR-31, miR-196a, miR-204, miR-206, miR-335
Pre-osteoblast	Proliferation	miR-23, miR-29, miR-34, miR-30, miR-31 miR-210 miR-218
Osteoblast	Matrix Maturation	miR-125b, miR-138, miR-637, miR-29c
Osteocytes	Mineralization	miR-23a~27a~24–2, miR-204, miR-205, miR-217, miR-133, miR-135

Role of miRs in mesenchmal stem cells and multiple bone cell types. Adapted from Lain and Stein *et al*, and Baglìo and DeVescovi *et al*.

**Fig 1:**
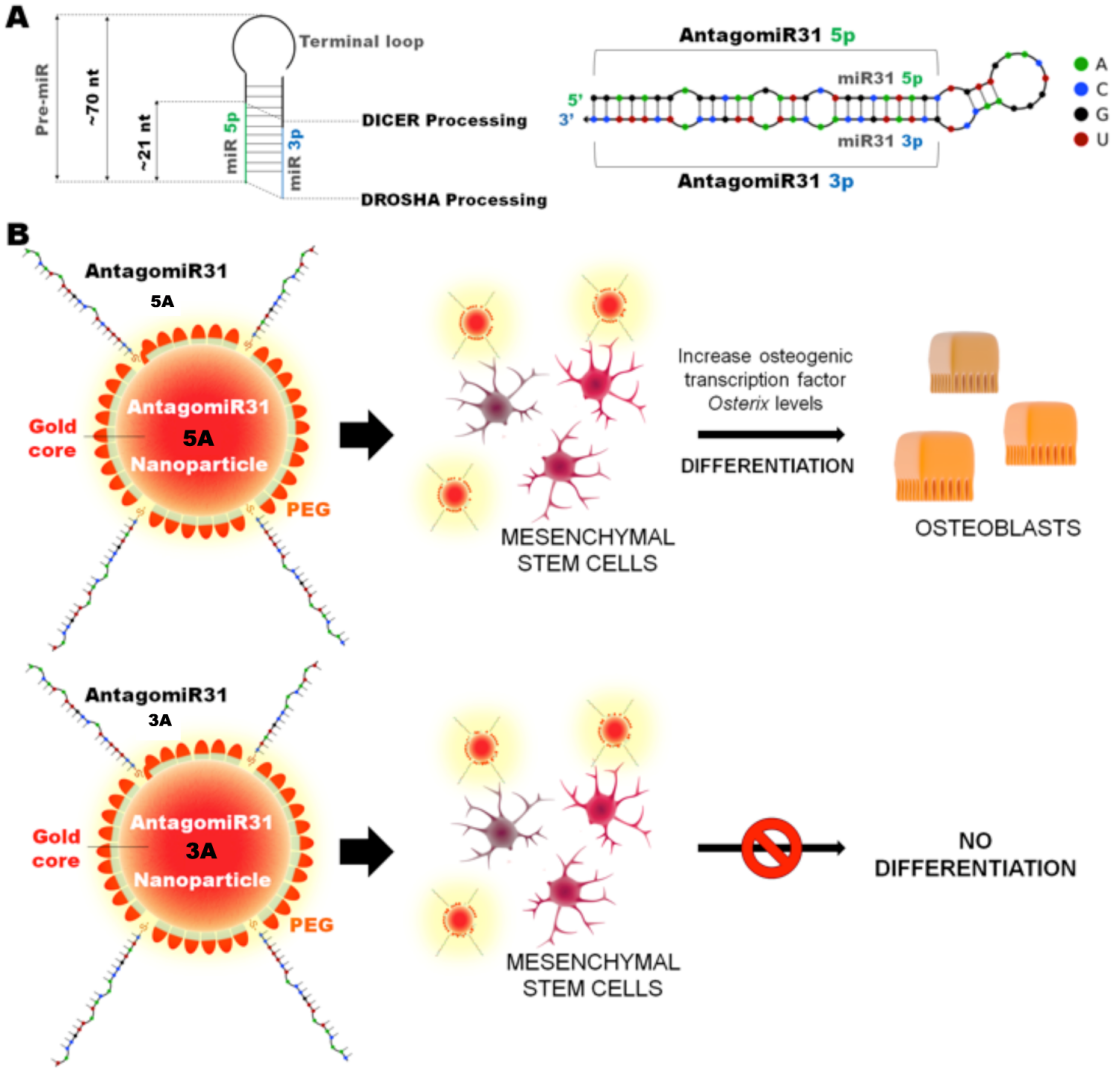
Overview of AntagomiR31 design, conjugation with nanoparticles and effect on cells. Schematic summary indicating (A) the 5’ and 3’ (5A and 3A) antagomiR design, prior to GNP functionalization, and (B) the sequence of events following incubation with mesenchymal stem cells. Note that only the 5A GNPs increased osterix expression and induced osteogenesis.

When comparing miR signatures in Table 1, it is noted that miR-31 has been implicated in osteogenesis in both MSCs and pre-osteoblastic cells. MiR-31 is hypothesised to act *via* suppression of a later-stage osteogenic transcription factor, osterix (downstream of runx2) [9, 12–17]. A recent report demonstrated changes in osterix when an osteosarcoma cell line, MG63 cells, (pre-osteoblastic phenotype) was treated with cholesterol-modified miR-31 sequences [12]. In the same year another group, using lipofectamine transfected cells, demonstrated that miR-31 expression was progressively decreased in human bone marrow derived stem cells undergoing osteogenesis, highlighting a potential role in differentiation [15, 16]. Meanwhile, overexpressing miR-31 reduced osterix, osteocalcin, and osteopontin protein expression without affecting RUNX2 protein levels, suggesting that miR-31 specifically influences downstream targets of RUNX2 and strongly support a role for miR-31 in osteogenesis [18, 19]. Due to the reported progressive loss of miR-31 in differentiating MSCs, we hypothesise that blocking miR-31 with antagomiRs at an early culture time point should allow an increase in osterix expression, encouraging osteogenesis. The exploitation of miRs and their RNA antagonists (antagomiRs) for widespread clinical treatment has, to date, been setback by technical issues, in particular their cellular delivery. Neither sequences can enter cells easily and are rapidly degraded *in vivo*; it is important therefore that a delivery vector is used, allowing delivery across the plasma membrane and to release the functional miR/antagomiR within the cell to allow interaction with the RNA induced silencing complex (RISC) [20]. Previous groups have delivered miRs and antagomiRs using cholesterol, however this limits the potency of the nanoconstruct to one antagomiR per delivery vector. Other groups have used lipofectamine to deliver miRs, however these liposomes tend to be cytotoxic and can leak their miR cargo. We believe gold nanoparticles (GNPs) are excellent candidates, as they have exceptionally low toxicity, many copies of the miR can be attached to the nanoparticle and GNPs have successfully delivered siRNA *in vitro* and *in vivo* [21, 22]. We therefore designed thiolated antagomiRs against miR-31, with both 5’ and 3’ reading direction (5A and 3A respectively), which were conjugated onto GNPs for delivery into cells. The efficacy of GNPs to deliver functional antagomiRs was initially verified in the human osteosarcoma cell line MG63 (pre-osteoblasts), prior to being assessed in primary human bone marrow MSCs. As summarised in Fig 1, whilst both 5A and 3A functionalised GNPs were successfully delivered into MSCs, only the 5A GNPs accelerated osteogenesis (Fig 1B).

## Materials and methods

Human MSCs were purchased from Promocell (cells are obtained from approved donor programs at associated medical centres). The cells were cultured for 24 hours before the addition GNP antagomiR treatments at 50nM (Table 2).

**Table 2:** GNPs used in this study.

	Nanoparticle Type	Acronym	AntagomiR (nM) on GNP
1	Au-5A-30% PEG	5A	965.35
2	Au-3A-30% PEG	3A	1058.53
3	Au-NS-30% PEG	NS	1123.81
4	Au-PEG-30% PEG	PEG	-

GNPs with corresponding antagomiR densities. Please note that 5A denotes the 5’ end of the antagonist sequence of miR-31, and 3A denotes the 3’ end of the antagonist sequence of miR-31. NS is a nonsense stand, non-targeting miRNA used as a negative control. The MSCs were cultured with antagomiR functionalised GNPs for 48 hours and 5 days.

### Synthesis and functionalisation of gold nanoparticles

All GNP synthesis was carried out as previously described [19].

#### Synthesis of citrate-gold nanoparticles

**Fig 2:**
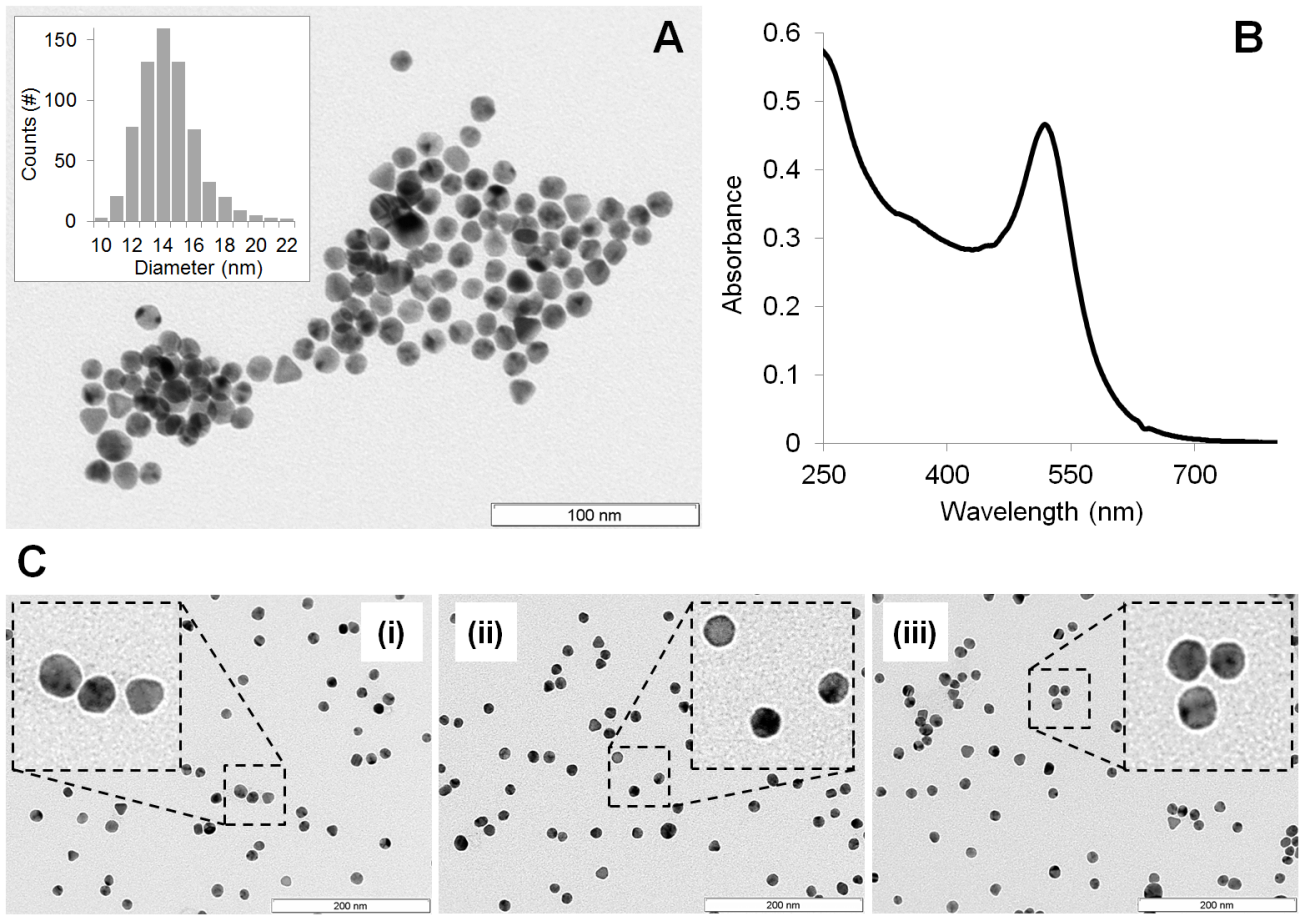
Gold nanoparticle characterisation. (A) TEM image of citrate-gold nanoparticles (scale bar = 100 nm). Inset: size distribution histogram showing an average diameter of 14.4±2.0 nm. (B) UV-Vis spectra of the synthesized GNPs with the characteristic surface plasmon resonance (SPR) peak at 519 nm. (C) TEM image of antagomiR-gold nanoparticles (scale bar = 200 nm) (i) = NS (ii) = 5A (iii) = 3As.

Gold nanoparticles, with an average diameter of 14.4±2.0 nm, were synthesized by the citrate reduction method. Briefly, 225 mL of 1 mM hydrogen tetrachloroaureate (III) hydrate (Sigma) (88.61 mg) dissolved in 500 ml of distilled water were heated to reflux while stirring. Then, 25 mL of 38.8 mM sodium citrate dihydrate (285 mg) were added and refluxed for additional 30 minutes with vigorous stirring and protected from light. The resulting red solution was cooled down and kept protected from light. Citrate capped GNPs were characterized by transmission electron microscopy (TEM) and UV-Vis spectroscopy (Fig 2). Poly (ethylene glycol) (PEG) functionalization of gold nanoparticles.

**Fig 3:**
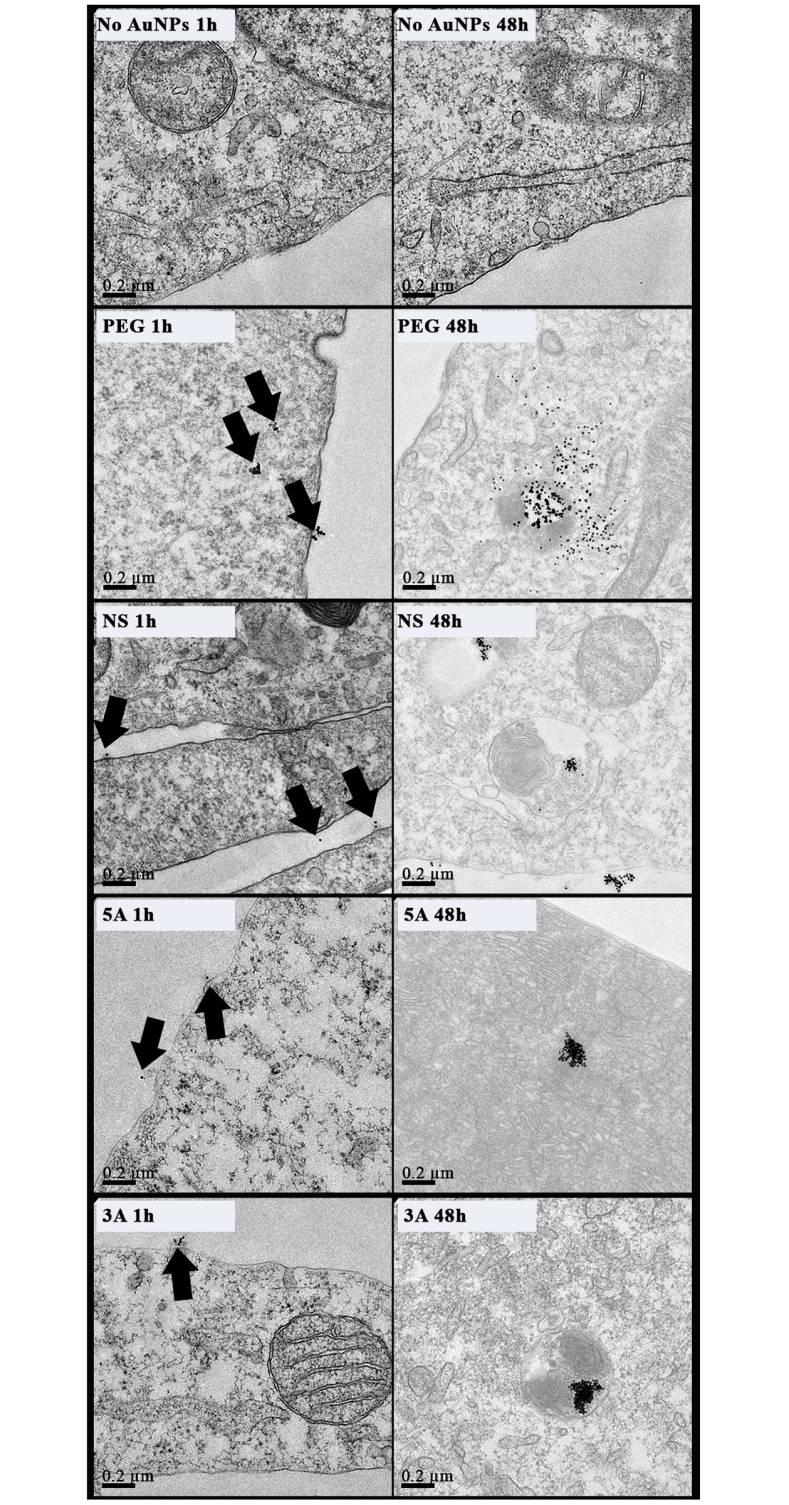
TEM images of MG63 cells treated with GNPs (50nM, 30% PEG) for 1 and 48 hours. Black arrowheads denote GNPs (n = 3 technical replicates; scale bar = 0.2μm).

Briefly, 10 nM of the GNP solution were mixed with 0.003 mg/mL of a commercial hetero-functional poly (ethylene glycol) (PEG) [O-(2-Mercaptoethyl)-O’-methyl-hexa (ethylene glycol), C_15_H_32_O_7_S, 356.48 Da, Sigma] in an aqueous solution of SDS (0.028%). Then, NaOH was added to a final concentration of 25 mM and the mixture incubated for 16 hours at room temperature. Excess PEG was removed by centrifugation (21.460 ×g, 30 min, 4°C), and quantified by a modification of the Ellmans’ Assay [19]; The excess of thiolated chains in the supernatants is quantified by interpolating a calibration curve set by reacting 200 μL of stock solution of the O-(2-Mercaptoethyl)-O’-methyl-hexa(ethylene glycol) in 100 μL in phosphate buffer 0.5 M (pH 7) with 7 μL 5 5’-dithio-bis(2-nitrobenzoic) acid (DTNB, Sigma) 5 mg/mL in phosphate buffer 0.5 M (pH 7), and measuring the absorbance at 412 nm after 10 minutes. The linear range (see Fig 3A and 3B) for the O-(2-Mercaptoethyl)-O’-methyl-hexa(ethylene glycol) chain obtained by this method is 0.0002–0.035 mg/mL (Abs412 = 26.229×[HS-PEG, mg/mL] + 0.0671). The number of exchanged chains is given by the difference between the amount determined by this assay and the initial amount incubated with the GNPs. There is a point at which the nanoparticle becomes saturated with a thiolated layer and is not able to take up more thiolated chains—maximum coverage per gold nanoparticle, i.e. 0.01 mg/mL of O-(2-Mercaptoethyl)-O’-methyl-hexa (ethylene glycol). The GNPs were functionalized with 0.003 mg/mL of O-(2-Mercaptoethyl)-O’-methyl-hexa (ethylene glycol) corresponding to 30% of PEG saturation of GNPs’ surface (200.16 ± 15.01 chains per nanoparticle) (S1 Fig).

#### Assembly of antagomiRs to PEGylated gold nanoparticles

Four sets of NP-antagomiRs were prepared using modified 2’-ACE (2bis(2-acetoxyethoxy)methyl) protected RNA oligonucleotides. 2’ACE is an orthoester group used to protect the 2-OH of RNA monomers from degradation and can used to store RNAs for extended periods and deprotected prior to use.

First, the RNA oligos were deprotected by adding 400 μL of 2’-Deprotection buffer (100 mM acetic acid, adjusted to pH 3.8 with TEMED), dissolving the oligo completely by vortexing and centrifuge 10 seconds. Then, the oligos were incubated at 60°C for 30 minutes and SpeedVac to dryness before reduction and purification of thiol groups. Briefly, the thiolated RNA oligonucleotides were suspended in 1mL of 0.1M dithiothreitol (DTT), extracted three times with ethyl acetate and further purified through a desalting NAP-5 column (Pharmacia Biotech) using 10 mM phosphate buffer (pH 8) as eluent. Following oligonucleotide quantification via UV/Vis spectroscopy, each RNA oligonucleotide was added to the GNP@PEG in a 100:1 ratio. AGE I solution (2% (w/v) SDS, 10 mM phosphate buffer (pH 8)) was added to the mixture to a final concentration of 10 mM phosphate buffer (pH 8), 0.01% (w/v) SDS, sonicated for 10 seconds using an ultrasound bath and incubated at room temperature for 20 minutes. Afterwards, the ionic strength of the solution was increased sequentially in 50 mM NaCl increments by adding the required volume of AGE II solution (1.5 M NaCl, 0.01% (w/v) SDS, 10 mM phosphate buffer (pH 8)) up to a final concentration of 10 mM phosphate buffer (pH 8), 0.3 M NaCl, 0.01% (w/v) SDS. After each increment, the solution was sonicated for 10 seconds and incubated at room temperature for 20 minutes. The solution was allowed to rest for additional 16 hours at room temperature. Then, the functionalized NP-antagomiRs were centrifuged for 20 minutes at 21.460 ×g, the oily precipitate washed three times with DEPC-treated H_2_O, and redispersed in the same buffer. The resulting NP-antagomiRs, as listed in Table 3, were stored in the dark at 4 °C until further use.

**Table 3:** Physical-chemical properties of the GNPs employed in the study.

NP Type	Acronym	SPR peak	Size (nm)^a^	Zeta-Potential (mV)^b^	PEG mol per particle	AntagomiR (nM) on GNPs
(reference only)	Unmodified NPs	519	14.4± 2.7	-19.2± 4.2	NA	NA
Au-PEG-30% PEG	PEG	521	18.5± 3.9	-25.2± 2.7	200.16 ± 15.01	NA
Au-NS-30% PEG	NS	523	39.75±1.2	-32.9± 1.4	200.16 ± 15.01	1123.81
Au-5A-30% PEG	5A	523	37.12±2.4	-34.3± 2.9	200.16 ± 15.01	965.35
Au-3A-30% PEG	3A	523	38.84±3.1	-32.3± 1.6	200.16 ± 15.01	1058.53

Please note that 5A denotes the 5’ end of the antagonist sequence of miR-31, and 3A denotes the 3’ end of the antagonist sequence of miR-31. NS is a nonsense stand, non-targeting miRNA, used as a negative control. SPR corresponds to the plasmon resonance, a method to determine NP aggregation.

^a^ Determined by Dynamic Light Scattering (DLS).

^b^ Nanoparticles analysed at a concentration of 2 nM in water in a total volume of 1 mL, with 0.1 M KCl.

Physical characterization of the NP-antagomiRs was performed by Dynamic Light Scattering (Zetasizer, Malvern), Zeta Potential (Zetasizer, Malvern), UV/Vis Spectroscopy and Transmission Electron Microscopy (see Table 3 and Fig 2).

### Cell culture: Osteosarcoma cell line (MG63s) and primary stem cells (MSCs)

The human osteosarcoma cell line MG63 (ATCC^®^ CRL-1427^™^) was employed in this study because of its innately high levels of miR-31. MG63s were expanded in Dulbecco’s Modified Eagle Medium (DMEM) growth medium with 10% FBS and 1% penicillin streptomycin and maintained at 37°C in 5% CO_2_ until ~90% confluent. The human MSCs (Promocell; isolated from human bone marrow samples) were expanded in DMEM supplemented with 10% FBS, 1% penicillin streptomycin, 1% 100mM Sodium Pyruvate and 1% Non-Essential Amino Acids, maintained at 37°C in 5% CO_2_ until ~70% confluent. All cells were seeded at a density of 1x10^4^ cells per ml for experiments, unless stated otherwise, and were cultured for 24 hours before the addition GNPs treatments at 50nM.

For induction of osteogenesis, DMEM for MSC culture described above was supplemented with dexamethasone (10nM), ascorbic acid (100μM) and β-Glycerophosphate (20mM).

### Toxicity

Cellular cytotoxicity was assessed by standard MTT (3-(4,5-Dimethylthiazol-2-yl)-2,5-diphenyltetrazolium bromide, a tetrazole) assay. Cells were seeded in a 96 well plate in triplicate. Following incubation with the relevant GNPs, cells were incubated in 100 μL of MTT solution (Sigma Aldrich; 5 mg/mL in PBS) for 60 minutes at 37°C. After which the MTT was aspirated off and 100 μL of dimethyl sulfoxide (DMSO, Fisher Scientific) was added for 10 minutes. The plate was subsequently read via spectrophotometry (Flurostar OMEGA at 550 nm).

### Cell internalisation of gold nanoparticles

#### Transmission electron microscopy (TEM)

Cellular uptake of GNPs was analysed via TEM. Cells were seeded at a density of 4x10^4^ cells per mL onto Thermanox coverslips (13mm diameter) and cultured to develop a confluent monolayer of cells. At this point the GNPs were added and cells further cultured for 1 and 48 hours. Cells were subsequently fixed with 1.5% glutaraldehyde in PBS for 2 hours, and washed for 10 minutes with PBS. Cells were then post-stained for 60 minutes with 1% osmium tetroxide in phosphate buffer, and postfixed in 1% buffer followed by 0.5% uranyl acetate for 1 hour, prior to being taken through alcohol dehydration increments and left in resin (propylene oxide Epon 812 resin mix (1:1)) overnight. Cell layers were captured in pure resin and cured overnight in an oven. Blocks were then cut into ultrathin sections, stained with 2% methanolic uranyl acetate and Reynolds lead citrate, and viewed under a Tecnai T20 (200 kV for cells).

#### Inductively coupled plasma mass spectrometry (ICP-MS)

Cellular uptake of GNPs was verified and quantified via ICP-MS. Cells were seeded (100 μL/well) in a 96 well plate and incubated for 24 hours. GNPs were added to cells for 48 hours. The media was transferred to 1 mL H_2_O (Millipore Ultra Pure) in a sample tube; 1 mL of concentrated 70% HNO_3_ was added to each vial and incubated overnight in a ~70°C water bath. Samples were made up to 50 mL with Millipore Ultra Pure water and analysed by ICP-MS at the SUERC, (East Kilbride, UK). The converted values for gold uptake were averaged (n = 3) and used for statistical analysis.

### Fluidigm analysis

Cells were seeded at 1 x 10^5^ cells per well on glass coverslips in a 24 well plate and allowed to adhere overnight. The GNP treatments were added to coverslips at a stated concentration and incubated for a specified time (control cells were incubated with media alone). After this, GNPs were removed and RNA was extracted using an RNeasy Mini Kit. Reverse transcription was performed using a SuperScript III Reverse Transcriptase (Invitrogen). Each qRT-PCR reaction contained 10ng of cDNA. The cDNA was pre-amplified with a pool of selected 100μM forward and reverse primers. After pre-amplification the samples were then treated with Exonuclease I treatment, to clean up any unincorporated primers. After Exonuclease I treatment the amplified samples were diluted in TE buffer (TEKnova, PN T0224). The samples were prepared as two technical replicates for every three biological replicates. Samples were pre-mixed with 2xSsoFast Evagreen Supermix (Bio Rad, PN 172–5211) and 20x DNA binding dye sample loading reagent (Fluidigm, PN 100–3738). Primers were prepared separately, with 100μM of forward and reverse primers to a final concentration of 5μM in loading reagent. The plate was primed using an IFC controller MX and loaded with samples on one side and primers on the other. Afterwards the plate was run using the BioMark Fluidigm system. Data was obtained by Fluidigm Real-Time PCR Analysis Software. Heatmaps were produced using the software PermutMatrix v.1.9.3.

#### Fluidigm analysis of osterix and related gene RNA levels

Analysis of MG63 cell RNA levels in response to antaogmir-31-GNP incubation was performed using the Fluidigm Biomark HD system. This system allows for automated PCR reactions to be carried out, using less samples and reagent, via a microfluidic design. A 48x48 array was used, allowing for multiple RNA targets to be assessed alongside several housekeeping genes. The targets used are detailed in S2 Table. Aside from the principal target osterix, these additional primers were selected based on their possible link to miR-31 through osteoblast-like pathways including RUNX2, the BMPs and SMADs (intracellular signalling proteins). From the qPCR data a heat map was created of the delta delta CT values: ((CT(target,untreated)−CT(ref,untreated))−(CT(target,treated)−CT(ref,treated)) from the samples normalised to multiple housekeeping genes, indicating increases or decreases in expression.

MG63s cells were grown at 1 x 10^5^ cells per well (MSCs at 1 x 10^4^) in a 24 well plate and allowed to adhere overnight. GNP treatments were added (50nM oligo on GNP surface) and incubated with the cells for 48 hours (control cells were incubated with media alone). After 48 hours, treatments were removed and RNA extracted using an RNeasy Mini Kit. Reverse transcription was performed using a SuperScript III Reverse Transcriptase (Invitrogen). Each qRT-PCR reaction contained 10ng of cDNA. The cDNA was pre-amplified with a pool of selected 100μM forward and reverse primers. After pre-amplification the samples were then treated with Exonuclease I treatment, to clean up any unincorporated primers. After Exonuclease I treatment the amplified samples were diluted in TE buffer (TEKnova, PN T0224). The samples were prepared as two technical replicates for every three biological replicates. Samples were pre-mixed with 2xSoFast Evagreen Supermix (Bio Rad, PN 172–5211) and 20x DNA binding dye sample loading reagent (Fluidigm, PN 100–3738). The stock primers were prepared separately, with 100μM of forward and reverse primers to a final concentration of 5μM in loading reagent. The plate was primed using an IFC controller MX and loaded with samples on one side and primers on the other. Afterwards the plate was run using the BioMark Fluidigm system. Data was obtained by Fluidigm Real-Time PCR Analysis Software. Heatmaps were produced using the software PermutMatrix v.1.9.3.

### In-cell western analysis of osterix protein levels

The level of osterix protein in MG63 cells and MSCs post GNP treatment was analysed via in-cell westerns. This allowed for both specific and high throughput analysis. Cells were seeded in a 96 well plate in triplicate, challenged with the GNPs for the desired time point (MSCs day 3, 5, 7 and 10; MG63s day 5), fixed, permeabilised (100ml PBS; 10.3g sucrose; 0.292g NaCl; 0.06g MgCl2 (hexahydrate); 0.476g Hepes and pH adjusted to 7.2, followed by the addition of 0.5ml Triton X)) and blocked 1% (w/v) Milk protein in PBS at 37°C for 1.5 hours. Samples were then co-incubated with primary antibodies (1:2000 mouse OSX and 1:5000 Cell tag 700) at 37°C for 1 hour. Following Tween washing, samples were subsequently co-incubated with secondary antibody (1:2000 donkey anti-mouse IR800CW, Licor, UK) at 37°C for 1 hour. All samples were finally washed three times in PBS/0.5%Tween (5 min/wash). The plates were imaged by scanning simultaneously at 700 and 800 nm with an Odyssey SA at 100 μm resolution, medium quality, focus offset of 3.53 mm, and an intensity setting of 7 for both 700- and 800-nm channels.

### Osteocalcin nodule formation by immunofluorescence

MSCs were seeded for 24 hours at 1x10^4^ cells per ml in DMEM. GNPs, 50nM, were added to cultures and left for 3 and 5 weeks. Media was changed every 3 days. Cells were fixed and permeabilised as for In-cell western. Non-specific binding sites were blocked by incubation with 1% (w/v) Milk protein in PBS at 37°C for 1.5 hours prior to incubating with Osteocalcin primary antibody (1:2000 mouse OCN sc-73464, Santa Cruz Biotechnology) for 1 hour at 37°C. Following washing, cells wre further incubated with anti-mouse Texas red secondary antibody (1:50) for 1 hour at 37°C, before washing and co-staining with DAPI mounting media (Vector Laboratories). All images were viewed using an Axiophot fluorescence microscope.

### Theoretical binding of antagomiR-31 sequences

Both the 5A and 3A antagomiR and the nonsense RNA sequences were run through the RNAhybrid programme developed by M. Rehmsmeier. RNAhybrid predicts the minimum free energy hybridization of two RNA sequences, allowing for miR target prediction. Hybridization is assessed by domain analysis with one sequence hybridized to the best fitting part of another [22].

### Statistics

Statistical analysis was performed in Graphpad using a one-way ANOVA with a Dunnett′s test. In all Figs * = *p* < 0.05, ** = *p* < 0.01, *** = *p* < 0.001 and **** = *p* < 0.0001. Two tailed T-Tests were performed where specifically mentioned, a Welch’s correction was used, * = *p* < 0.05, ** = *p* < 0.01.

## Results and discussion

MiRNAs are becoming recognised as crucial regulatory molecules in MSC lineage commitment and terminal differentiation[23]. A decrease in miR-31 has recently been reported during osteogenesis, linked to osterix expression [9, 12–17, 24]. Herein, we identify a potential role for miR-31 in induction of osteogenesis using GNPs as a delivery platform for antagomiRs targeted against MiR-31.

### Gold nanoparticle synthesis, functionalisation and cell internalisation

GNPs were synthesised and functionalised as described by Conde *et al* (2012) [21]. GNPs were characterised for size *via* TEM and ultraviolet-visible spectroscopy (Fig 2A & 2B) and the concentration of PEG molecules on the GNP surface was calculated (S1 Fig). Thiolated antagomiR sequences were produced (Dharmacon; S1 Table), with either the 5’ or 3’ reading direction (denoted as 5A and 3A respectively) and attached to the GNPs. Analysis demonstrated disperse GNPs within a narrow size range (Fig 2C).

In summary, the panel of GNPs employed in this study, with associated acronyms, physical properties and PEG chain densities, is detailed in Table 3. All GNPs were assessed for potential cytotoxicity *via* MTT assay, with no adverse effects noted (S3 Fig).

**Fig 4:**
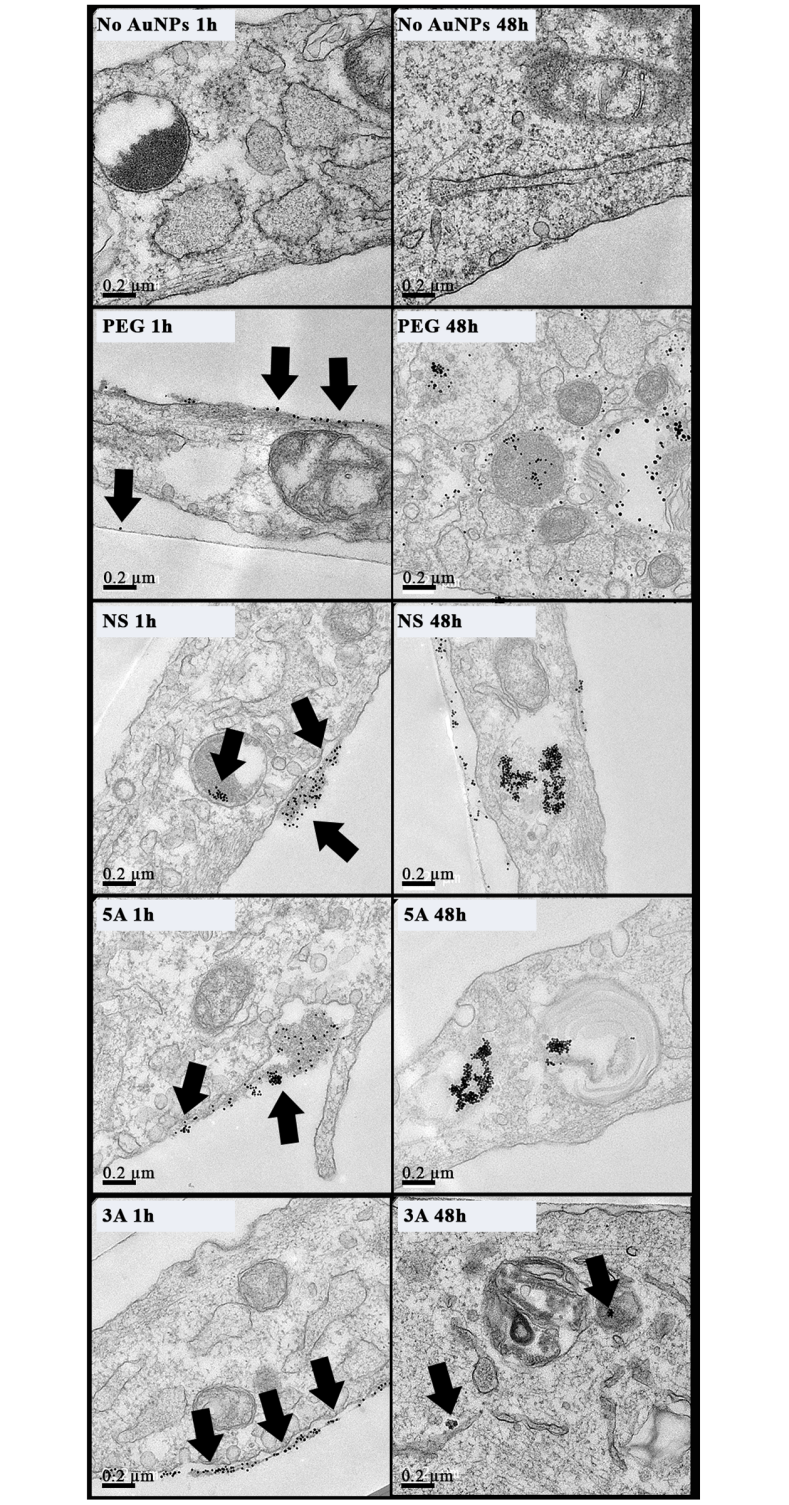
TEM images of MSCs cells treated with GNPs (50nM, 30%) for 1 and 48 hours. Arrows indicate GNP clusters (n = 3 technical replicates; scale bar = 0.2μm).

The amount of GNPs within the cells was quantified after 48 hours using inductively coupled plasma mass spectrometry (ICP-MS) to determine elemental gold levels in lysed cell samples. All four GNP types were verified within MG63 and MSCs, with increased internalization in MSCs and slight variations between the GNP species noted (S2 Fig). Following ICP-MS, cross-sectional imaging by TEM was further used to qualitatively analyse the cell internalisation and the intracellular location of the GNPs after both 1 hour and 48-hour incubations. For both the MG63s and MSCs, at 1 hour GNPs were mainly evident at the cell periphery (Figs 3 and 4), whilst at 48 hours the NPs were clearly evident within the cell, mainly packaged into endosomes (Figs 3 and 4). The metabolic activity of both cell types was not adversely affected following GNP internalization (S3 Fig).

### Osterix and related gene expression in response to GNP treatment

**Fig 5:**
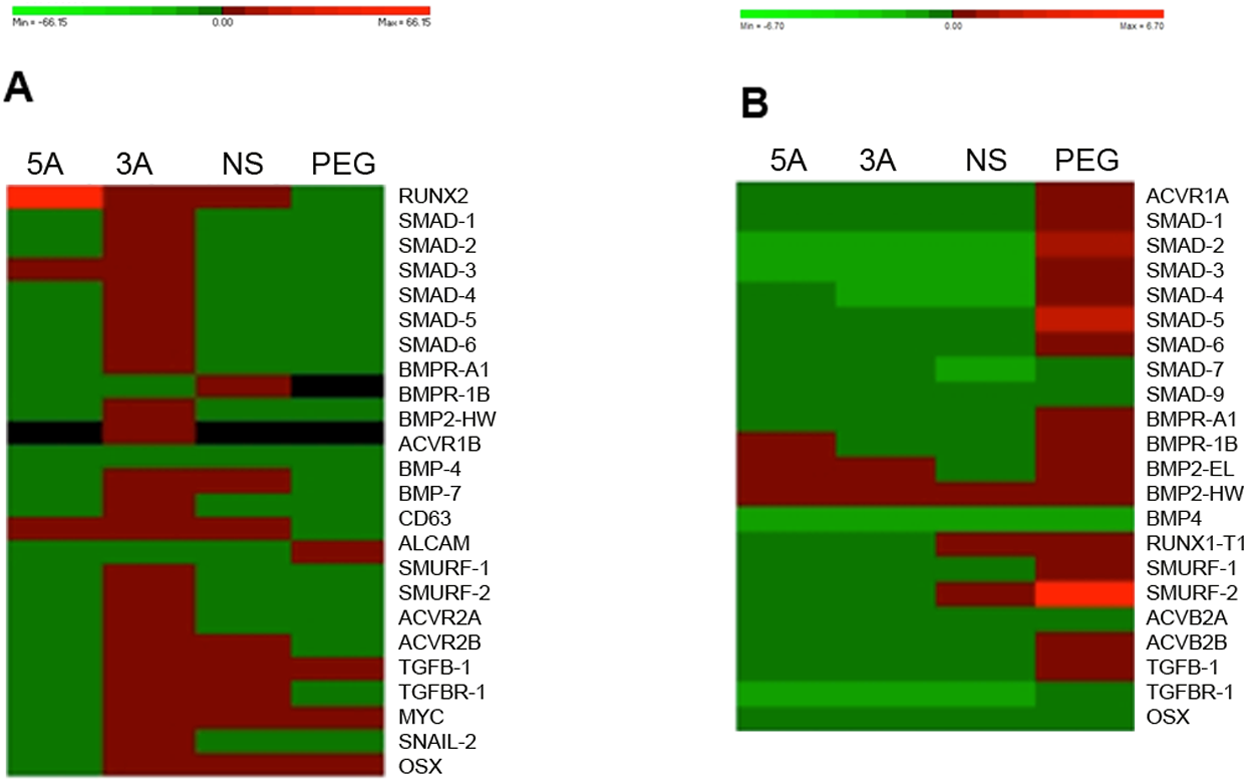
Fluidigm analysis. Heat-Map of (A) MG63s and (B) MSCS treated with the GNPs for 48 hours (green reflects a decrease in expression, whilst red reflects an increase). The data was analysed using PermutMatrix and ordered into hierarchy based on expression similarity.

Changes in osterix RNA may not be expected, as the delivered antagomiR interaction would be post-transcriptional and occur outside the nucleus in the RISC complex [15]. However, any evidence for changes in osterix (or related gene expression) may indicate a feedback response. From the raw fluidigm data with both cell types after 48 hours GNP incubation, a hierarchical analysis was used to group the GNP species together based on the similarity of the CT values (Fig 5).

Fluidigm analysis at the RNA level generally demonstrated a decrease in osterix, aside from the 3A antagomiR and MG63 cells (Fig 5A). Further, transcriptional analysis indicated, when observing changes in other gene expression profiles, a cellular shift away from proliferation towards differentiation with the antagomiRs. This was most notable with the osteo-committed MG63s, where a 5A-mediated reduction in C-Myc expression alongside an increase in runx2 and SMAD3 indicated that the cells were perhaps reducing proliferation to focus on enhancing commitment along the osteoblast lineage (Fig 5A). Also with the more primitive MSCs, BMPR-2 and BMP-R1A, which are co-involved in the very early cell signalling pathways for osteogenesis [22], transcript changes may suggest early stages of osteo-commitment (Fig 5B).

The RNA analysis also demonstrated a host of up- and down-regulations for a range of genes in response to GNPs (i.e. the NS samples in Fig 5), suggesting a general cell reaction to the GNPs at the RNA level unrelated to the antagomiRs. This is not surprising as the addition of NPs to the culture media, and subsequent internalization, would be expected to stimulate a variety of cellular responses. There is very little literature in this regard, as most reports using NP delivery platforms focus primarily on assessing the functional delivery of the cargo. However, recent work has noted non-target-related gene effects when using siRNA delivered to cells *via* GNPs [25].

Furthermore, it was also notable that the 3A GNPs up-regulated most genes, in direct comparison to the 5A GNPs, that down-regulated everything apart from RUNX2, SMAD3 (both involved in the osteoblast phenotype) and CD63 (HOP-26 related to MSC phenotype); i.e. sensible targets for osteo-commitment. It could be postulated that the 3A antagomiR may be non-specifically up-regulating multiple RNAs *via* a more promiscuous binding, whilst 5A’s more conservative binding (i.e. binding to one target very strongly and very weakly to any other sequence) might enable a more targeted action.

Overall, considering that miR events are largely post-transcriptional [26–28], alongside many gene changes, we did observe potential indications of osteo-commitment at the gene transcriptional level.

### Increase in osterix protein expression with antagomiR-GNP treatment

**Fig 6:**
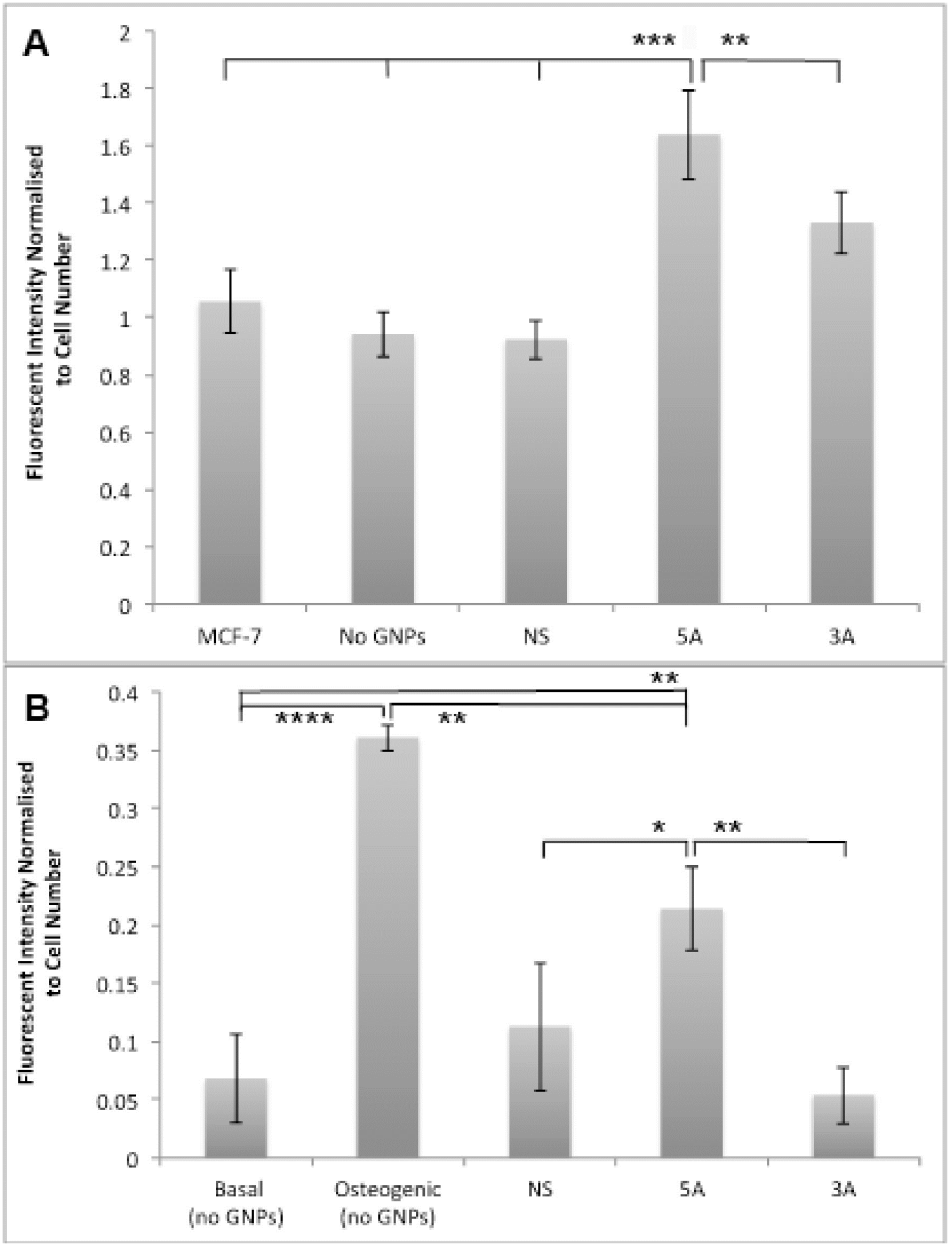
In cell western data of osterix protein levels. (A) at day 5 in MG63s after 48 hours incubation with GNP treatments normalised to cell number and to PEG GNPs, and in MCF-7 cells (n = 6; error bars denote standard deviation); (B) at day 7 in MSCs after 48 hours incubation with GNP treatments normalized to cell number and to PEG GNPs. (n = 6; error bars denote standard error, **** = p<0.0001; *** = p<0.001; ** = p<0.01 compared to no GNPs).

In-cell western was carried out to study changes in Osterix at the protein level after both cell types were incubated with GNPs for an initial 48 hours. osterix was significantly increased in MG63 cells with both the 5A and 3A treatments, compared to cells treated with no GNPs, PEG or NS controls (Fig 6A). The breast cancer cell line MCF-7 was used as a negative control as it is known to contain low levels of osterix; no difference was noted between these cells and the MG63 cells alone, or those incubated with the NS and PEG GNPs. This suggests that MG63 cells also maintain a basal osterix expression level, which was significantly increased in response to the antagomiR functionalised GNPs.

Osterix protein levels in MSCs were assessed day 7 post GNP treatment. The sequence-modified GNPs (5A, 3A and NS) were subsequently normalised to PEG NPs and compared against control MSC cultures, grown either in basal DMEM media or osteogenic media (Fig 6B). The osteogenic media samples demonstrated a highly significant increase in osterix when compared to all the other conditions, as expected. However, the 5A GNPs also significantly increased osterix expression when compared to the basal media, the NS sequence and the 3A GNPs. The 3A GNPs did not record any change in osterix levels.

### Osteocalcin nodule formation in MSCs following early treatment with antagomiR-GNPs

**Fig 7:**
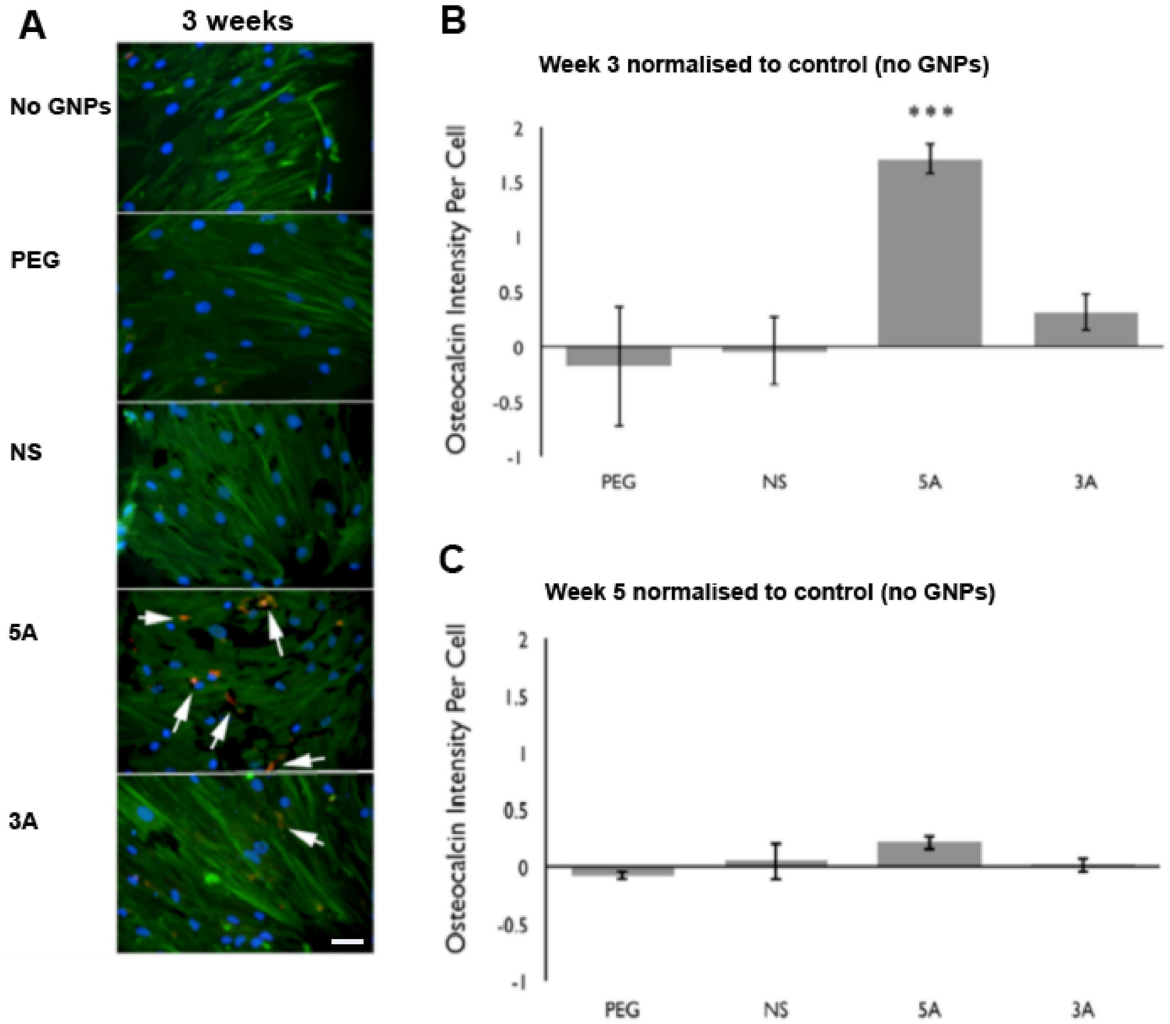
Osteocalcin nodule formation in MSCs. (A) Representative MSC images from GNP treatment after 3 weeks. Staining indicates actin (green), nucleus (blue) and osteocalcin nodules (red). Scale bar = 5μm (white arrows highlights osteocalcin staining). (B&C) Semi-quantification of osteocalcin staining in MSCs after treatment with GNPs for 3 and 5 weeks (B and C respectively). All treatments have been normalised to control cells treated with no GNPs. (n = 3, error bars denote SEM *** = *p* < 0.001).

As there was a significant increase in osterix protein expression in response to the 5A and 3A GNPs with the MG63 cells and a rapid initial increase observed in osterix in response to the 5A GNPs with the MSCs, this leads to the question of whether this increase is sufficient to drive changes in downstream expression of bone-related proteins. To assess this, MSCs were cultured with the panel of GNPs for 48 hours, and subsequently cultured for a further 3 weeks before being stained for osteocalcin, a late osteogenic cell marker. Fig 7A indicates osteocalcin staining with both the antagomiR-GNP treatments, most notably with 5A. Consequent image analysis of multiple samples verified highly significant increases in osteocalcin with the 5A treatment (Fig 7B). This trend remained, but was far less evident by 5 week of culture, suggesting that the 5A antogomir treatment addresses the speed of osteogenesis rather than level *per se* (Fig 7C).

MSC differentiation is a major research area. *In vitro* studies have shown that chemical induction [13, 29] or substrate (e.g. topography, stiffness) driven induction [30–32] tend to follow fairly rigid temporal-differentiation guidelines originally described by Stein and Lian [33]. These studies illustrate a rapid slowing of growth followed by induction maxima for RUNX2 at ~day 5, osterix at ~day 10, alkaline phosphatase at ~day 14, osteocalcin at ~day 21 and finally mineralisation at ~day 28. All of these studies have focused on maximising the number of cells entering this timecourse (% osteogenesis) [22].

It is therefore interesting to note that in this new study, the use of antagomiRs appears to have stimulated osterix as an earlier time-point than would normally be expected, at ~day 7 rather than ~day 10. This then drives highly significantly increased osteocalcin expression at week 3. By week 5, control samples appear to have caught up. This demonstrates that, compared to the traditional approaches described above, our new antagomiR based approach can be used to induce early osteogenic onset rather than total osteogenic commitment.

### AntagomiR sequence directionality influences differentiation

Whilst the key results in this paper demonstrate a clear link between miR-31 suppression and accelerated osteogenesis, the study also revealed that efficacy varied depending on the antagomiR sequence directionality (i.e. 5A denotes the 5’ end of the antagonist sequence of miR-31, and 3A denotes the 3’ end). A complication when reading miRNA literature is the possible variation in the sequence origin; this study therefore compared antagomiR sequences with 5’ (denoted 5A) and 3’ (3A) directionality. Although both the 5A and 3A antagomiRs are antagonists of miR-31, the two species are from different sections of the miR-31 sequence. Data analysis clearly shows that the two sequences behave differently despite their origin; 5A being more efficient. With this noted, questions about the sequence binding are raised.

**Fig 8:**
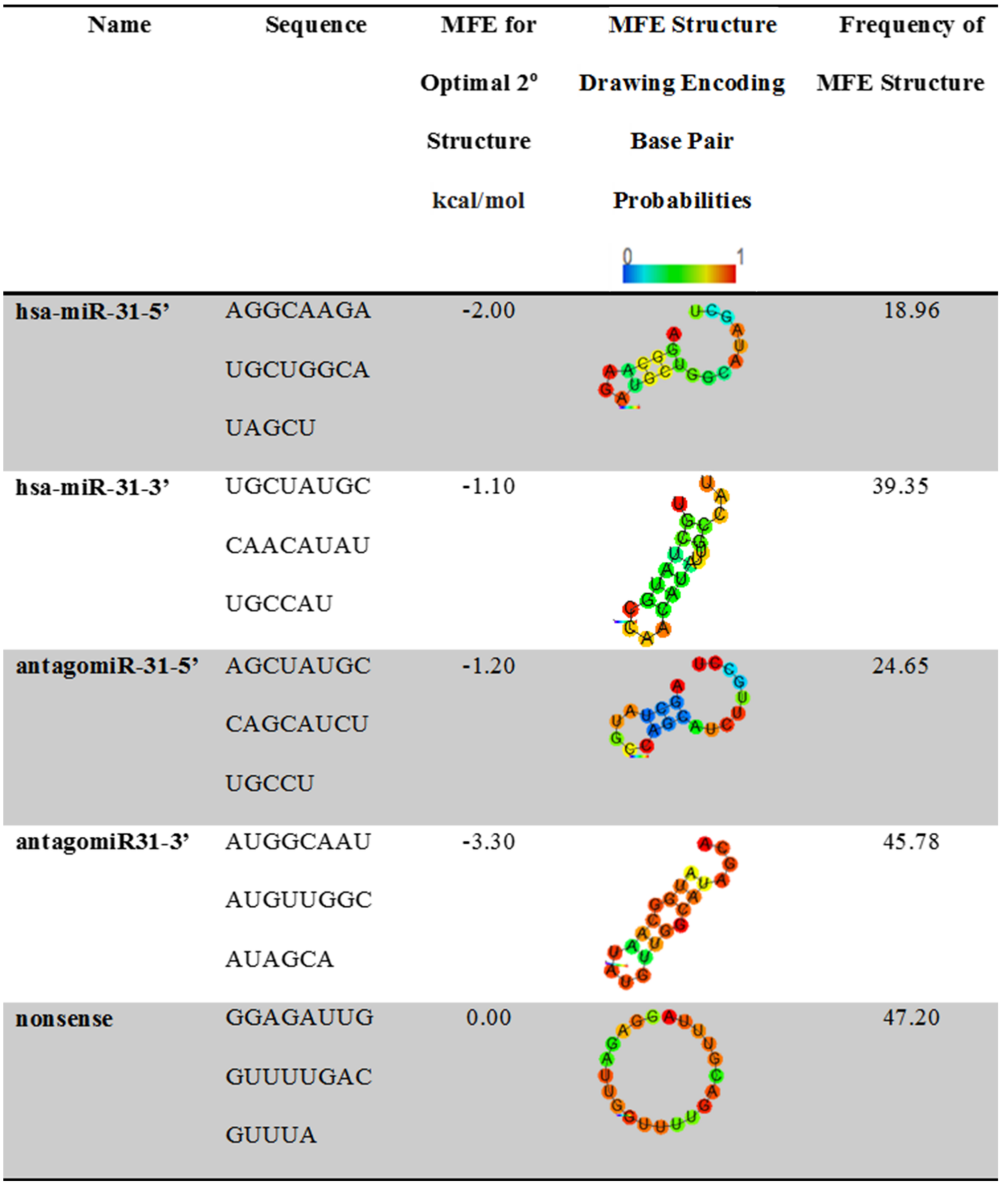
Predicted structures of the single stranded RNA sequences. Structures are based on the minimal free energy (MFE) method (an established method to predict RNA structure). Complimentary regions are evaluated to predict the most energetically stable molecule. The stability of the structure is given in kcal/mol, the more negative the value equates to a more stable structure (‘hsa’ denotes species; Homo sapiens).

**Fig 9:**
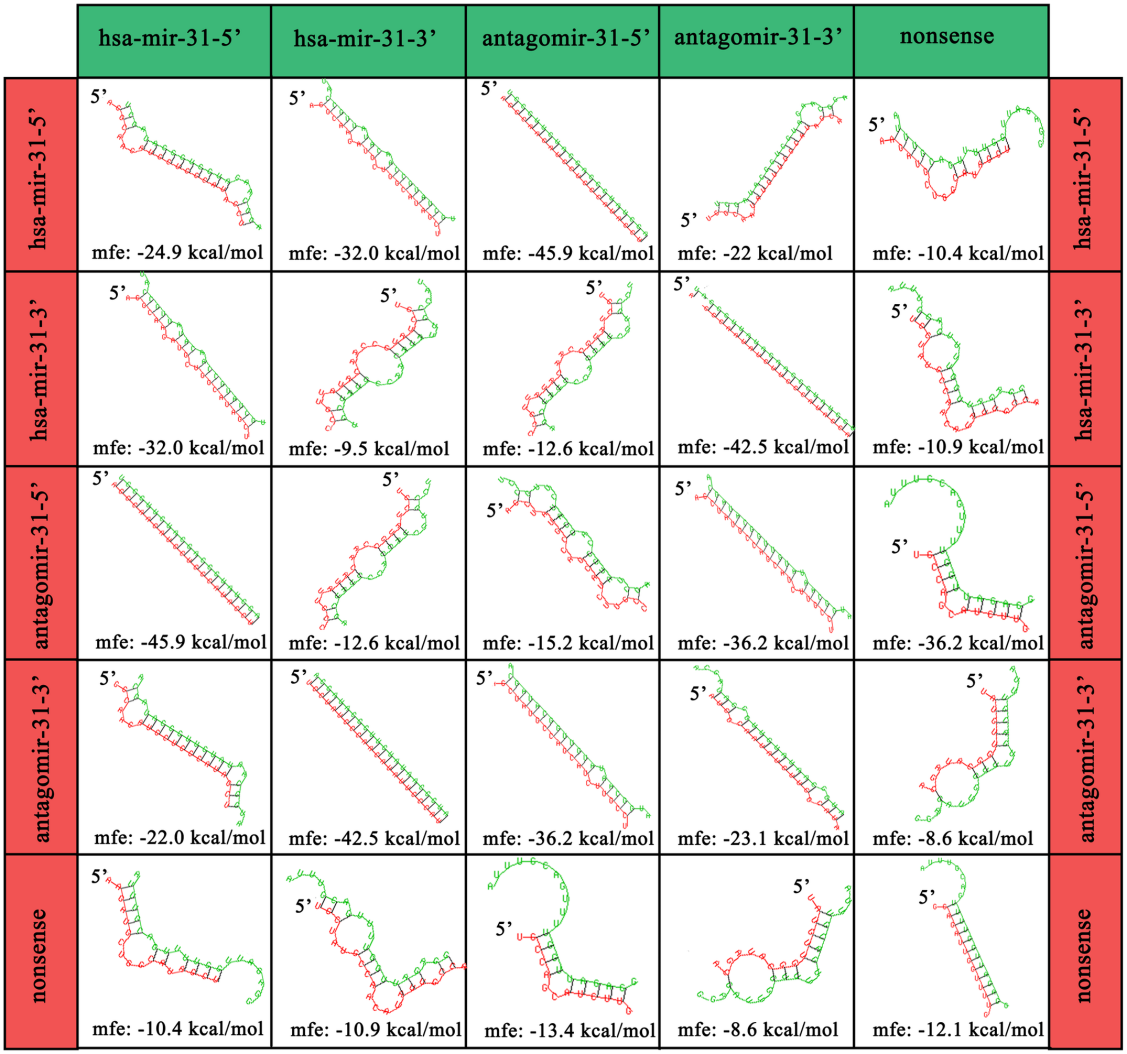
MiRNA binding. Sequence geometries are based on base pairing and minimum free energy using RNAhybrid. The stability of the structure is measured in kcal/mol (i.e. the energy required to break the structure). The greater the energy needed, the more stable the structure. Linearity infers stability, whilst loops indicate incompatibility and lack of binding. The antagomiRs and their corresponding miRNA sequence form the strongest and most stable structure.

To investigate the antagomiR binding potentials the RNAhyrbid program (designed by Rehmsmeier *et al*, 2004) was used to determine the type and strength of bond that both antagomiRs would form with the corresponding and opposing strand [34]. The predicted structure for the sequences is shown in Fig 8, whilst the binding potential results are shown Fig 9. The binding energies of both antagomiRs for their corresponding miR-31 (miR-31-5’ with 5A and miR-31-3’ with 3A) target sequence indicated strong bond formation that required ~ -40 kcal/mol to dissociate. The antagomiR 3’ sequence (i.e. 3A) was predicted to form a bond with the opposing 5’ sequence of miR-31 (-22.9 kcal/mol), whilst the 5’ antagomiR sequence (5A) was predicted to form a weaker structure with the opposing 3’ miR-31 sequence (-12.6kcal/mol). As expected, the nonsense strand’s ability to bind to miR-31 was even weaker.

This differential between binding energies of the 5A and 3A with the different miR-31 stands could be a reason for the range of responses observed. A report by Chan *et al* in 2013, found a range of concordant and disconcordant responses to miR-31 by shifting the sequence by one nucleotide [35]. This change was reported to repress dicer activity (a major component of the RNAi pathway), whilst the nonshifted sequence could not. Thus, other mechanisms apart from seeding region may induce a drastic change in properties by subtle variations in sequence length and position. This improved perspective could lead to better design of miR therapeutics.

The different efficiency of 5A and 3A could also be due to differences in the structure of the primary RNA sequences, which, based on the minimum free energy method, are shown in Fig 8. MiR-31 5’ and the corresponding 5’ antagomiR (5A), when single stranded, form structures with large open unbound sequences of ~9 bp in length. This open, unbound section could allow for easier binding with targets, resulting in more efficient attenuation.

## Conclusion

The manipulation of MSC differentiation has been extensively studied to date, with particular advances in chemical and physical induction. Research over the last decade has demonstrated that miR regulation of MSC differentiation may be key to unlocking new techniques to influence differentiation. In this regard, several recent papers demonstrated a progressive loss of miR-31 during osteogenesis; we therefore hypothesized that blocking miR-31 with antagomiRs at an early culture time point should permit an increase in MSCs committing to an osteoblastic lineage. In this manuscript we have shown that by using GNPs as delivery platforms for antagomiR sequences against miR-31, we can indeed increase osterix expression in MSCs, encouraging osteogenesis. Such delivery systems hold promise for conditions such as age-derived osteoporosis; endothelial-secreted microvesicular miR-31, which increases with age, has been reported to inhibit osteogenesis, thus inhibition of miR-31 may reverse this effect [36]. In addition, very few antagomiR studies focus on the origin of the sequence employed and the possible differences of having a sequence that targets the passenger strand over the guide strand might produce; our data clearly indicates that this is important to consider in future work.

## Supporting information

S1 Fig GNP saturation with thiolated PEG. (A) Absorbance spectra of DTNB after reaction with the thiolated PEG. (B) Standard calibration curve for PEG chains, whose concentration can be calculated via the following equation Abs412 = 26.229× [HS-PEG, mg/mL] + 0.0671. (C) Variation of the excess of PEG thiolated chains as a function of the initial concentration in the incubation with 10 mM GNPs. The dashed vertical line indicates the 100% saturation, i.e. the PEG concentration above which no more PEG can be bonded to the GNPs surface. (D) Ratio between non-aggregated (at 520 nm) and aggregated NPs (at 600 nm) of GNPs after functionalization with increasing amounts (0–0.035 mg/mL) of thiolated PEG.
(TIF)

S2 Fig ICP-MS analysis. (A) MG63s and (B) MSCs treated with GNPs (50nM, 30%) for 48 hours. All GNP species were found within both cell types. Each lysate has an n = 3, error bars denote SD.
(TIF)

S3 Fig MTT analysis. (A) MG63 cells and (B) MSCs treated with each GNP (50nM oligo, 30% PEG) type for 48 hours (PEG, NS, 3A, 5A) (n = 3; error bars indicate SD).
(TIF)

S1 Table AntagomiR sequences. S1 Table showing the oligomer sequences used for GNP-antagomiR functionalization. GC % relates to the melting temperature; the greater the GC content the higher the melting temperature. AntagomiR-31 5’, is designed to bind with the corresponding miR-31 5’ sequence. The same principle relates to antagomiR-31 3’, which binds with perfect complementarity to the miR-31 3’ sequence.
(PDF)

S2 Table List of fluidigm primers used in this study. Primer list used for fluidigm analysis, detailing the gene function and the forward and reverse sequences used. Those with * indicate housekeeping genes.
(PDF)
