# Supplementary figures and images for: Nanoparticle-antagomiR based targeting of miR-31 to induce osterix and osteocalcin expression in mesenchymal stem cells
Source: PLoS One. 2018 Feb 14;13(2):e0192562. doi: 10.1371/journal.pone.0192562 (PMC5812622; doi:10.1371/journal.pone.0192562)

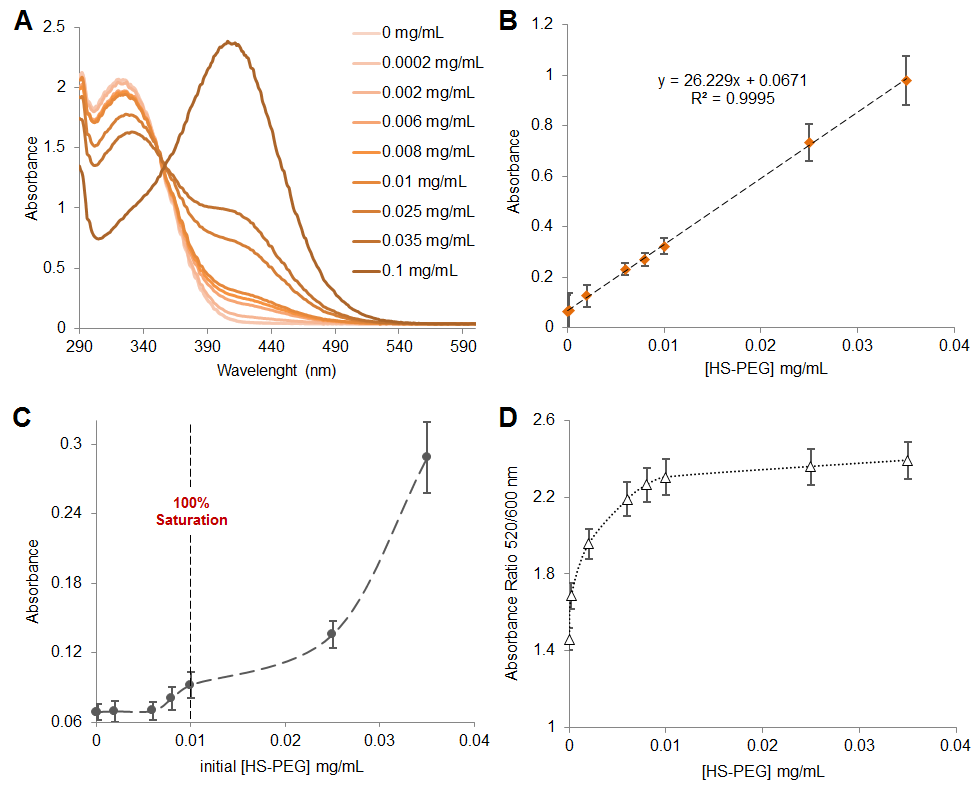

Supplement: S1 Fig — (A) Absorbance spectra of DTNB after reaction with the thiolated PEG. (B) Standard calibration curve for PEG chains, whose concentration can be calculated via the following equation Abs412 = 26.229× [HS-PEG, mg/mL] + 0.0671. (C) Variation of the excess of PEG thiolated chains as a function of the initial concentration in the incubation with 10 mM GNPs. The dashed vertical line indicates the 100% saturation, i.e. the PEG concentration above which no more PEG can be bonded to the GNPs surface. (D) Ratio between non-aggregated (at 520 nm) and aggregated NPs (at 600 nm) of GNPs after functionalization with increasing amounts (0–0.035 mg/mL) of thiolated PEG. (TIF) [file pone.0192562.s001.tif]

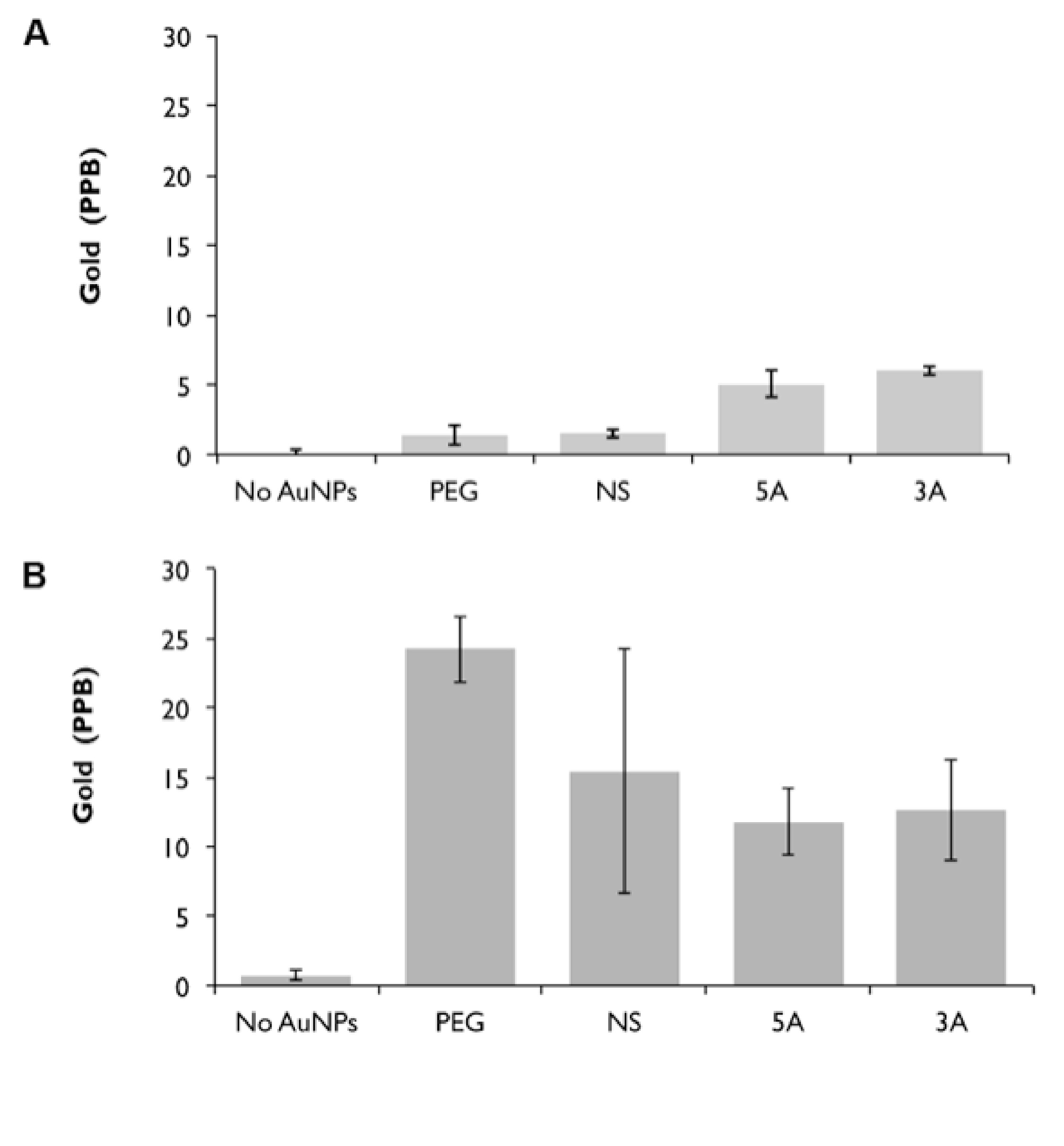

Supplement: S2 Fig — (A) MG63s and (B) MSCs treated with GNPs (50nM, 30%) for 48 hours. All GNP species were found within both cell types. Each lysate has an n = 3, error bars denote SD. (TIF) [file pone.0192562.s002.tif]

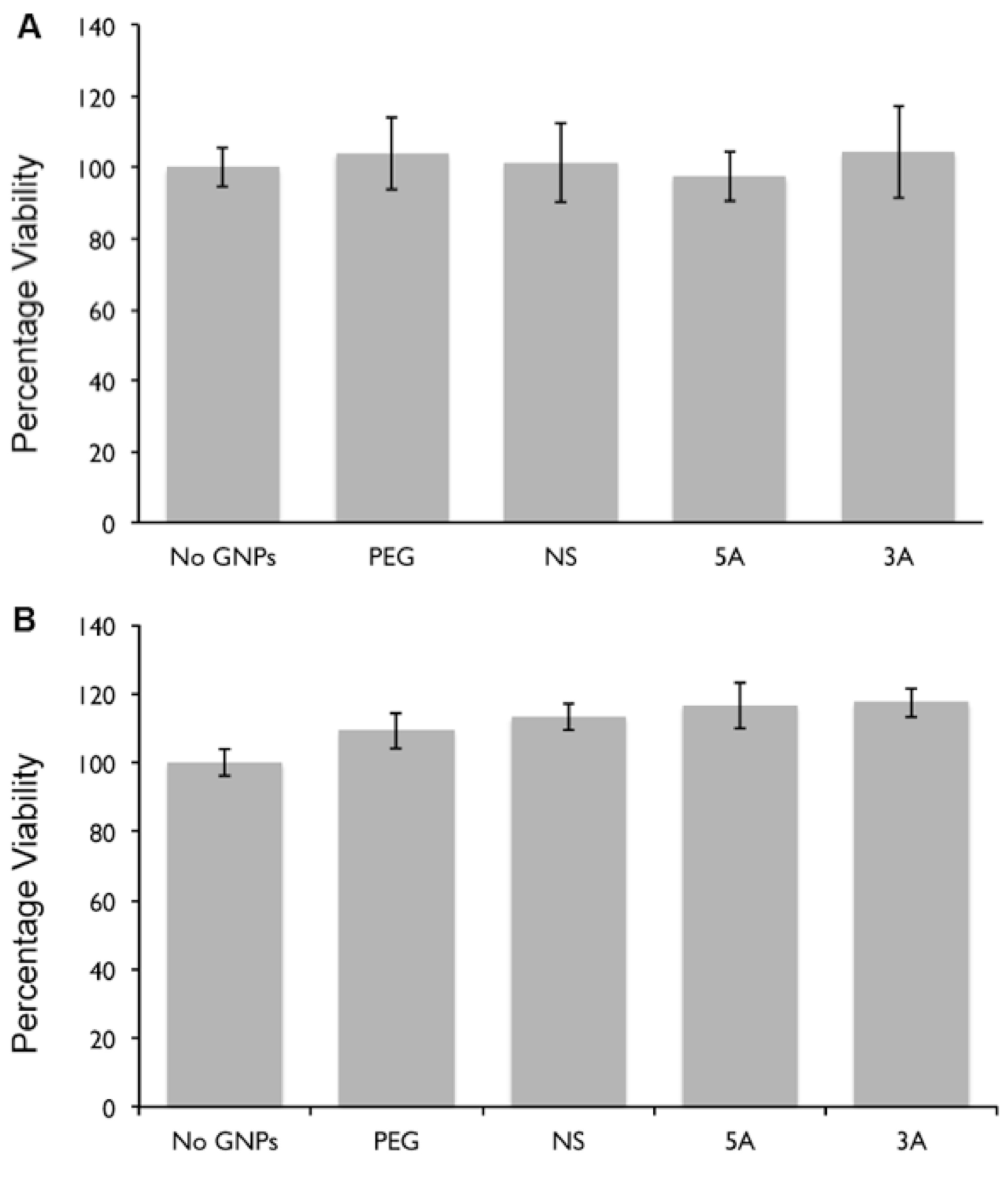

Supplement: S3 Fig — (A) MG63 cells and (B) MSCs treated with each GNP (50nM oligo, 30% PEG) type for 48 hours (PEG, NS, 3A, 5A) (n = 3; error bars indicate SD). (TIF) [file pone.0192562.s003.tif]
