# Supplementary material for: Nanoparticle-antagomiR based targeting of miR-31 to induce osterix and osteocalcin expression in mesenchymal stem cells
Source: PLoS One. 2018 Feb 14;13(2):e0192562. doi: 10.1371/journal.pone.0192562 (PMC5812622; doi:10.1371/journal.pone.0192562)
Supplement: S1 Table — showing the oligomer sequences used for GNP-antagomiR functionalization. GC % relates to the melting temperature; the greater the GC content the higher the melting temperature. AntagomiR-31 5’, is designed to bind with the corresponding miR-31 5’ sequence. The same principle relates to antagomiR-31 3’, which binds with perfect complementarity to the miR-31 3’ sequence. (PDF) [file pone.0192562.s004.pdf]

| AntagomiRs | Sequences | GC% | Melting Temperature (ºC) |
|---|---|---|---|
| antagomiR non-targeting | S–S•GGAGAUUGGUUUUGACGUUUA | 38 | 48.5 |
| AntagomiR-31 5’ | S–S•AGCUAUGCCAGCAUCUUGCCU | 52 | 54.4 |
| antagomiR-31 3’ | S–S•AUGGCAAUAUGUUGGCAUAGCA | 41 | 51.1 |
