# Supplementary material for: Nanoparticle-antagomiR based targeting of miR-31 to induce osterix and osteocalcin expression in mesenchymal stem cells
Source: PLoS One. 2018 Feb 14;13(2):e0192562. doi: 10.1371/journal.pone.0192562 (PMC5812622; doi:10.1371/journal.pone.0192562)
Supplement: S2 Table — Primer list used for fluidigm analysis, detailing the gene function and the forward and reverse sequences used. Those with * indicate housekeeping genes. (PDF) [file pone.0192562.s005.pdf]

Primer	Function	Sequence (5' to 3')
**β-Actin***	Housekeeping Gene	Forward GTGGGCCGCCCTAGGCACCAG<br>Reverse CACTTTGATGTCACGCACGATTTC
**RUNX2**	Transcription factor associated with osteoblast differentiation	Forward CAGCAGCAGCAACAGCAG<br>Reverse GGCGATGATCTCCACCAT
**ACVR1A**	Binds BMPs to then form complexes with SMADs	Forward GCCAAGGGGACTGGTGTAAC<br>Reverse GAGAATAATGAGGCCAACCTCCA
**SMAD1**	Mediates signals with BMPs by receptors	Forward GCTGCTCTCCAATGTTAACCG<br>Reverse CACTAAGGCATTCGGCATACAC
**SMAD2**	Mediates signals with BMPs by receptors	Forward CCACGGTAGAAATGACAAGAAGG<br>Reverse GATTACAATTGGGGCTCTGCAC
**SMAD3**	Mediates signals with BMPs by receptors	Forward GTCTGCGTGAATCCCTACCAC<br>Reverse GGGATGGAATGGCTGTAGTCG
**GNB2L1***	Receptor for activated C kinase 1	Forward TCCATACCTTGACCAGCTTG<br>Reverse GCAGATTGTCTCTGGATCTC
**SMAD4**	Common mediator SMAD, enhances SMAD signaling	Forward GGGTCAACTCTCCAATGTCCAC<br>Reverse GTCACTAAGGCACCTGACCC
**SMAD5**	Mediates signals with BMPs by receptors	Forward TGGGTCAAGATAATTCCCAGCCT<br>Reverse GGCTCTTCATAGGCAACAGGC
**SMAD6**	Inhibitory SMAD, block R-SMAD activation	Forward CTCCCTACTCTCGGCTGTCT<br>Reverse AGAATTCACCCGGAGCAGTG

**SMAD7**	Inhibitory SMAD, block R-SMAD activation	Forward CCATCACCTTAGCCGACTCT<br>Reverse CCAGGGGCCAGATAATTCGT
**SMAD9**	Mediates signals with BMPs by receptors	Forward CTTATCATGCCACAGAAGCCTCT<br>Reverse GCTCCTCGTAACAAACTGGTCG
**BMPR1A**	Bone morphogenetic protein receptor, type IA (CD292)	Forward ACGCCGGACAATAGAATGTTGTC<br>Reverse GAGCAAAACCAGCCATCGAATG
**TWY1***	Wyosine biosynthesis protein	Forward ATTGTCATCAAGACGCAGGGC<br>Reverse GTTGCGAATCCCTTCGCTGTT
**BMPR1B**	Bone morphogenetic protein receptor type-1B (CDw293)	Forward GGTTCAGACTTCTGCTGATTCAT<br>Reverse CGCAAAAGCATGTTATCAAGG
**BMP2-EL**	Osteoinductive cytokine, linked to hedgehog pathway and TGF beta signaling	Forward CTTCTAGCGTTGCTGCTTCC<br>Reverse AACTCGCTCAGGACCTCGT
**BMP2-HW**	Osteoinductive cytokine, linked to hedgehog pathway and TGF beta signaling	Forward AGACCTGTATCGCAGGCACT<br>Reverse CCACTCGTTTCTGGTAGTTCTTCC
**BMPR2**	Serine/threonine receptor kinase that binds bone morphogenetic proteins	Forward AGCCTCTCACACCCACTCC<br>Reverse GCAGAACAACCGTGAGAGG

**ACVR1B**	Binds ACVR2A or ACVR2B to recruit SMADS 2/3.	Forward GACATTGCCCCGAATCAGAGG<br>Reverse GCCCGAGGGCATAAATATCAGC
**BMP4**	BMP4 is found in early embryonic development	Forward CAGCACTGGTCTTGAGTATCCT<br>Reverse AGCAGAGTTTTCACTGGTCCC
**CYCR***	Bacterial housekeeping gene with adenylate cyclase activity	Forward ACTGCGGGAAGGTCTCTACTT<br>Reverse GGGTGCCATCGTCAAACTCTA
**BMP7**	Phosphorylates SMAD1 and SMAD5	Forward CAGGCCTGTAAGAAGCACGA<br>Reverse TGGTTGGTGGCGTTCATGTA
**BMP10**	Involved in the trabeculation of the heart	Forward ACCCACCAGAGTACATGTTGG<br>Reverse GCCCATTAAAACTGACCGGC
**Nestin**	A type VI intermediate filament, mainly a nerve stem cells marker	Forward GCTCAGGTCCTGGAAGGTC<br>Reverse AAGCTGAGGGAAGTCTTGGA
**CD63**	A transmembrane protein, signaling cell growth, development and motility	Forward CCCTTGGAATTGCTTTTGTT<br>Reverse TATTCCACTCCCCCAGATGA
**ALCAM**	(CD1660) A transmembrane glycoprotein, mediating adhesions	Forward TTCCAGTCCCTCTACTCAGAGC<br>Reverse GCTAAGAAGGACTCGCAGGA

**Osterix**	A master regulatory transcription factor for osteogenesis	Forward TGGGCTCCCAACACTATTTC<br>Reverse GGGAAGACTGAAGCCTGGA
**UBE2D2***	Ubiquitin-conjugating enzyme E2 D2	Forward CCATGGCTCTGAAGAGAATCC<br>Reverse GATAGGGACTGTCATTTGGCC
**RUNX1T1**	A zinc finger transcription factor, that blocks hematopoietic differentiation	Forward ATCACAACAGAGAGGGCCAA<br>Reverse CTGCAGGTTTCACTCGCTTT
**SMURF1**	E3 ubiquitin-protein ligase regulates SMAD proteins	Forward ATGCAGTTCGTGGCCAGATA<br>Reverse CAGGCCCGGAGTCTTCATAC
**SMURF2**	E3 ubiquitin-protein ligase regulates SMAD proteins	Forward GACAGGATCCTCTCGAGTGC<br>Reverse AGCTTTCATAGGGTGGAATGTCT
**INHBA**	Inhibin beta A, a differentiation factor	Forward AAGTCGGGGAGAACGGGTAT<br>Reverse GGTCACTGCCTTCCTTGGAA
**ACVR2A**	Forms a dimer to activate TGF-Beta pathway.	Forward ACCATGGCTAGAGGATTGGC<br>Reverse GCCAACCCAAAGTCAGCAAT
**ACVR2B**	Forms a dimer to activate TGF-Beta pathway. Binds with a 3-4 higher affinity than ACVR2A	Forward CTGCAACGAACGCTTCACTC<br>Reverse CAGGACGATGAGGGAAAGGC
**RNF20***	E3 ubiquitin-protein ligase BRE1A	Forward GGTGTCTCTTCAACGGAGGAA<br>Reverse TAGTGAGGCATCATCAGTGGC

**TGFB1**	Cytokine of the TGF-beta superfamily, that controls cell growth, differentiation and proliferation	Forward CGACTCGCCAGAGTGGTTATC<br>Reverse GTTATCCCTGCTGTCACAGGAG
**TGFBR1**	The receptor for TGFB1, that transduces further signalling	Forward CGTTCGTGGTTCCGTGAGG<br>Reverse TAATCTGACACCAACCAGAGCTG
**TRAF6**	Tumor necrosis factor receptor, transduces signaling for MAP3K pathways	Forward CGCACTAGAACGAGCAAGTGA<br>Reverse GCCACACAGCAGTCACTTTCA
**SNAIL2**	Regulator of osteoblast differentiation	Forward TCCTTCCTGGTCAAGAAGCA<br>Reverse GGTATGACAGGCATGGAGTA
**Vimentin**	A type III intermediate filament protein expressed in MSC	Forward GGAGAAATTGCAGGAGGAGA<br>Reverse TGCGTTCAAGGTCAAGACGT
**IL-08**	Chemokine involved in immune response and angiogenesis	Forward GTGTGAAGGTGCAGTTTTGCC<br>Reverse GTGGTCCACTCTCAATCACTC
**B2M***	MHC class I molecule present on all nucleated cells	Forward TTGTCTTTCAGCAAGGACTGG<br>Reverse ATGCGGCATCTTCAAACCTCC
**CathepsinB**	Marker for cell death and inflammation	Forward TGTGTATTCGGACTTCCTGC<br>Reverse TTAAAGAAGCCATTGTCACCC
**CathepsinD**	A lysosomal aspartyl protease	Forward GGTGCTCAAGAACTACATGG<br>Reverse ATTCTTCACGTAGGTGCTGG

**CathepsinG**	Protease found mainly in immuno cells	Forward AACAGATACACTCCGAGAGG<br>Reverse ACGACTTTCCATAGGAGACG
**CathepsinL**	Lysosomal endopeptidase enzyme initiating protein degradation	Forward GACTCTGAGGAATCCTATCC<br>Reverse CTTAGGGATGTCCACAAAGC
**CathepsinS**	lysosomal cysteine protease	Forward GCGTCATCCTTCTTTCTTCC<br>Reverse CCAGCTGTTTTTCACAAGCC
**GAPDH***	Enzyme catalyzing a step of the glycolysis pathway	Forward TCAAGGCTGAGAACGGGAA<br>Reverse TGGGTGGCAGTGATGGCA
